# Research on optimization of train routing problem for railway hubs considering passenger demand

**DOI:** 10.1371/journal.pone.0351229

**Published:** 2026-06-15

**Authors:** Yidong Wang

**Affiliations:** China Railway Economic and Planning Research Institute CO., LTD, Beijing, China; Beijing University of Technology, CHINA

## Abstract

The railway hub with multiple stations has been more common with the expansion of the railway network. It is imperative to investigate the utilization of multiple stations to meet the travel demand of urban residents. This paper focuses on railway hubs and abstracts the division of labor among multiple stations as an optimization of train routing problem. A mixed integer linear programming model is constructed to minimize the total cost of trains and passengers, with the number of stops, capacity of railway lines and station tracks, and passenger demand of traffic zones considered as constraints. A case study based on a multi-station railway hub is implemented to evaluate the effectiveness of the optimization model. Compared with the other two scenarios, the total cost of the optimization results is reduced by 0.1% and 2.7%. Meanwhile, the proposed optimization scheme also achieves a more balanced and efficient utilization of railway line and station track capacity. Furthermore, the research also proposes several operationally feasible recommendations for railway department to support making division of labor scheme for railway hubs.

## 1. Introduction

With the continuous construction of railway lines, many cities have established multiple railway stations, and railway hubs with multiple stations have become typical [1]. Multi-station hubs not only alleviate the operational pressure of existing passenger stations but also provide more travel options for residents within the city. However, as the number of trains gradually increased after the line was opened, the disparity in workloads among the various stations within the hub also gradually became apparent, leading to an uneven distribution of capacity. This imbalance restricts the overall transportation efficiency of the hub, increases the time for trains to pass through railway hubs and also wastes the capacity of facilities, which is not conducive to reducing the operational costs of railway transportation.

Meanwhile, with the continuous expansion of urban scale, the travel demand of residents has grown rapidly and shown a diversified development trend of multi-regions, high efficiency and high quality [2]. However, affected by urban space and division of labor schemes, some passengers cannot go to the nearest station in large cities, they even have to travel a long distance to catch the train to their destination, which limits the quality of railway passenger transport services and causes the loss of passenger flow.

Therefore, how to optimize the division of labor among stations based on the configuration of hub facilities and the travel demand of urban residents has become a core issue in enhancing the transportation efficiency of railway hubs. This research abstracts the division of labor problem in multi-station railway hubs into a train routing problem, we optimize the division of labor in multi-station railway hubs by optimizing the train routes and stop schemes within the hub. Additionally, we further introduce travel zones to quantify the travel demand of residents within the city, ensuring that the division of labor scheme can take into account the travel demand of residents in different areas. On the one hand, it can provide optimization strategies for railway transportation departments to enhance the transportation efficiency of hubs, and activate the potential of the railway network. On the other hand, it can also offer development suggestions for passenger transport departments to improve the level of transport services, which is conducive to expanding the market share of railway transport.

The remainder of this research is organized as follows. Section 2 summarizes the related literature. Section 3 proposes the problem description. Section 4 formulates the mathematical model. Section 5 gives a case study consisting of several experiments to test the optimization model. Section 6 provides concluding remarks for railway hubs and further research.

## 2. Literature review

In the railway transportation, the optimization of trains travel path from origin to destination is typically formulated as the Train Routing Problem (TRP). The core objective of this problem is to minimize both travel time and operational cost. Depending on the path type and its hierarchical level, TRP could be further categorized into macroscopic and microscopic levels. As one of the most prevalent optimization problems in this field, TRP has generated a large number of studies.

At the macroscopic level, the railway network is abstracted as a graph comprising stations as nodes and railway lines as edges, with the primary focus on determining travel paths for trains. This level is mainly applied to line planning and timetable optimization at the network level. The optimization objective typically involves minimizing the total travel time and operating costs across all trains, subject primarily to line capacity constraints. While, at the microscopic level, the detailed topology within stations is further refined, including tracks, switches, and signaling equipment, with emphasis on arrival and departure routes. This level is mainly applied to track allocation and station operations optimization, where the objective is to minimize travel time and route occupation time, subject primarily to platform track capacity constraints and throat area operational constraints [3,4]. As this study focuses on railway passenger hubs comprising multiple stations, specifically addressing train routing and stopping patterns within such hubs. Accordingly, the literature review is structured around two main parts: macroscopic train routing problem and division of the hub.

For train routing problem, early studies on train routing optimization primarily focused on single-track railways, and subsequently expanded to double-track railways before further extending to complex railway networks [5]. This progression reflects a transition in research objects from simplified line structures toward more realistic and complicated network topologies, meeting the growing complexity of modern railway systems [6]. Carey et al. [7,8] developed a model, algorithms, and strategy for routing trains with varying speeds and stopping patterns on a unidirectional double-track railway in a previous paper, and then extended to more general and complex rail networks encompassing line choices, and station platforms. Lee et al. [9] proposed an optimization heuristic algorithm that integrates train pathing and train timetabling, with the capability to solve practically problems. A distinctive feature of this heuristic algorithm is its modeling flexibility, which improved the conventional assumption of fixed parameters, thereby better reflecting real-world operational conditions. Sun et al. [10] proposed a multi-objective optimization model, which considered the average travel time, the energy consumption, and the user satisfaction. And an improved genetic algorithm was designed to solve the train routing problem. Li et al. [11] proposed a bi-level model for simultaneously obtaining the optimal plan of passenger assignment and train routing considering the total train travel time, the average capacity utilization rate, the total travel time of passengers, and the average train attendance rate for all sections. And a particle swarm approach was adopted to solve the problem. Zhou et al. [12] constructed a directed graph to represent the composite travel network encompassing both train services and passenger flows, through which train operational choices, passenger travel choices, and their interdependent constraints could be simultaneously captured. And a mixed-integer linear programming approach was developed to jointly solve the line planning and timetabling problem. Wang et al. [13] addressed the challenge of long-distance cross-line operations under resource constraints by proposing an integrated train line planning and timetabling approach that modified train operation zones. A bi-objective model was formulated to minimize deviations of same-line trains from ideal timetables while maximizing direct cross-line origin-destination services. Zhou et al. [14] addressed complex railway networks comprising both unidirectional and bidirectional tracks, and proposed a train-based Lagrangian relaxation decomposition method for the simultaneous optimization of passenger train routing and timetabling. Zhang et al. [15] proposed a unified integer linear programming (ILP) model with two types of binary decision variables coupled through cross-resolution consistency constraints. The line planning component satisfied passenger demand via ILP, while cyclic timetabling was formulated as a multi-commodity network flow model with track capacity constraints. Wang et al. [16] proposed a mixed integer programming model based on an event-activity network framework, and the model objective was set to minimize the deviation of main-line trains from their ideal timetables while maximizing the frequency of direct services for cross-line passengers.

For division of the hub problem, it is defined as the rational planning of the types and numbers of passenger trains to be received and dispatched at each station within a railway hub, based on the coordinated utilization of all transportation resources in the hub, with the objective of minimizing train operational costs under the capacity constraints of various equipment and facilities. Caimi et al. [17] proposed a model predictive control framework as a dispatching assistant for complex central station areas. Liao et al. [18] advanced railway capacity estimation beyond conventional infrastructure-centric approaches by integrating both infrastructure and vehicle resources. Capacity was defined through a timetable saturation method, formulated as an integer programming model on a hybrid time–space network to maximize overall transportation performance. Shi et al. [19] formulated a multi-commodity flow model on a two-layer time–space network to capture the joint optimization of train platforming and shunting with service scheduling in railway hubs. The model incorporated the spatiotemporal coupling between train arrival, departure operations and shunting services, aiming to mitigate operational conflicts, reduce shunting workload, and achieve balanced resource utilization across stations and depots. Park et al. [20] investigated line planning problems with multiple patterns, analyzed the computational complexity, and developed a column generation algorithm for solution. The effectiveness of the approach was validated, offering novel modeling insights for improving passenger service efficiency and network coverage. Pellegrini et al. [21] addressed the real-time railway traffic management problem by proposing a mixed-integer linear programming with fine-grained infrastructure representation. Khaled et al. [22] proposed an optimization model under disruptive scenarios, aiming to minimize system-wide total cost, including classification time at yards and travel time along links. The optimization was to determine the optimal number of trains, their routes, and associated blocks, subject to various capacity and operational constraints at rail links and yards. Sama et al. [23] compared two selection strategies: a tactical-level approach that pre-screened alternative routings offline after timetabling using historical data, and an operational-level approach that selected routings online before real-time dispatching based on up-to-date perturbation information. Szymula et al. [24] proposed a Railway Network Vulnerability Model (RNVM) from the dual perspective of passenger flows and train operations, assessing system vulnerability by identifying critical link combinations. An integrated solution framework was developed that synergized column generation, row generation, and mixed integer linear programming, enabling the simultaneous generation of critical links, reassigned passenger flows, rerouted train paths, and adjusted timetables. Yu et al. [25] addressed the coordination of heavy and empty vehicle flows in railway hubs by developing an integrated optimization model and designing a logistic chaos map-enhanced artificial bee colony algorithm. Zhou et al. [26] addressed flow assignment for stage plan formulation across multiple marshalling stations. An optimization model was constructed with the objective of minimizing total dwell time, taking into account disassembly and assembly capacity at marshalling stations, transfer operation capacity between stations, connection time limits for inbound and outbound trains, and heterogeneous full workload constraints.

In summary, the existing researches of train routing problem mainly focus on either macroscopic line planning or microscopic track allocation, which are difficult to be directly applied to division of the hub problem. In addition, the existing researches on hub division mainly took the perspective of railway transportation enterprises, with relatively insufficient consideration of passenger travel demand. Based on this, the main contribution of this paper is stated as follows: According to the layout of lines and stations in railway hubs, we combine macroscopic line planning with microscopic track allocation to detail the train routes in railway hubs. Meanwhile, we also set up travel zones to quantify the travel demand of urban residents, and incorporate them into the optimization model to improve the quality of the division plan.

## 3. Problem description

**Fig 1:**
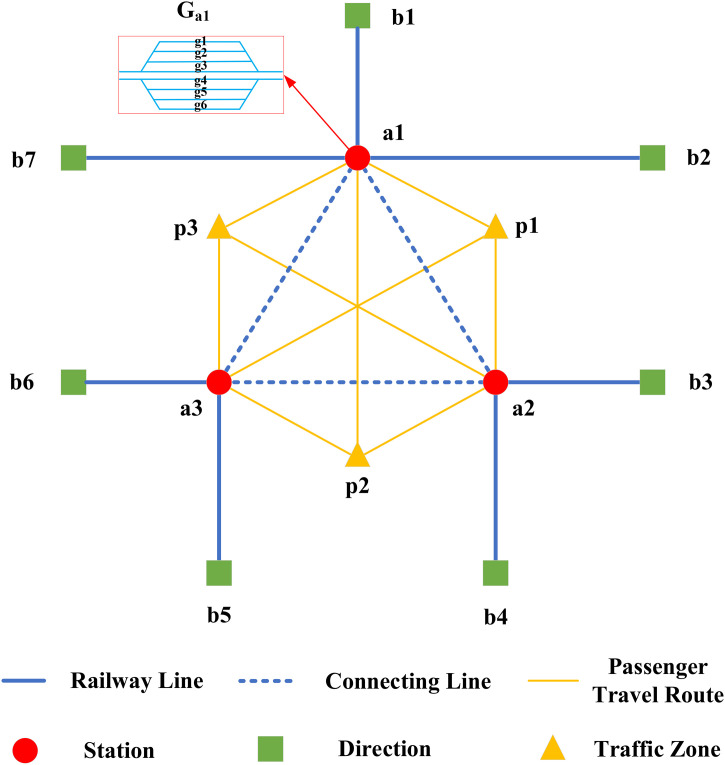
The network structure of the railway hub.

The issue of division of labor among passenger stations within a railway hub essentially involves the rational allocation of the types and quantities of trains. Furthermore, this study takes a single train as the basic object to make the scheme more flexible, unlike previous studies that took the flow of trains with the same origin and destination as the basic object. Therefore, the division of labor problem can be regarded as the train routing problem in the hub, where the train route indicates the running path and the operation track, and the railway hub network is constructed as shown in Fig 1.

The details of the railway hub are presented as follows:

(1) Set of nodes

The set of nodes V includes: (a) railway stations A, indexed by a, (b) connecting directions B, indexed by b, (c) traffic zones *P*, indexed by p. Specifically, the connecting direction indicates the direction of a railway line, and the traffic zone indicates the travel demand of residents going to certain directions.

(2) Set of arcs

The set of arcs E includes: (a) the arcs $E_{\text{1}}$ between railway stations A and connecting directions B, as shown by the blue arcs and the dotted arcs, which refer to the double-track railway lines, (b) the arcs $E_{\text{2}}$ between railway stations A and traffic zones *P*, indicate the passenger travel routes and are shown by the yellow arcs.

(3) Set of tracks

The set of tracks *G* refers to the tracks of all stations, indexed by *g*. The tracks of station a are represented as $G_{a}$.

(4) Capacity of railway lines and tracks

The capacity of arc $(i,j)\in E_{\text{1}}$ refers to the number of trains that can pass through the railway line within a certain period of time. The capacity of track g∈G refers to the number of trains that can be operated on the track within a certain period of time.

Based on the railway hub network, the division of labor can be represented as the train routing problem as follows: given (a) the network structure of the railway hub, including the layout of the railway lines and the details of the stations, (b) the capacity and time of the railway lines and tracks, (c) the number of various types of trains, (d) the travel demand of passengers, (e) the passing time of railway lines, tracks and passenger travel routes, (f) the unit time cost of trains and passengers, we aim to minimize the total cost of trains and passengers.

## 4. Modelling

### 4.1 Assumptions

Without loss of generality, the following assumptions are made to simplify the formulation:

(1) During the decision-making period, only passenger trains stopping at the hub are considered, which includes the departure trains, arrival trains, and passing trains that stop at the hub. Since non-stop trains do not perform operations within the hub and have a relatively small impact on the capacity of lines and tracks, they are not taken into account.
(2) The operation time of different tracks within the same station is various, and the difference is directly proportional to the distance between the tracks. Specifically, the further the track is from the main track, the longer the train route within the station, and thus the longer the operation time.
(3) Only the departure passenger flow is considered. The traffic zones set in this research mainly represent the external travel demand of residents within the hub and do not include the arrival and transferring passenger flow. The modeling complexity of the transfer passenger flow is relatively high and the train schedule needs to be introduced into the optimization model [10,27,28].Considering the overall scale of the arrival and transfer passenger flow are relatively small compared to the departure passenger flow, we think its impact on the overall solution result will not be significant.
(4) During the decision-making period, the capacity of other facilities within the railway hub can meet the requirements, such as the capacity of the EMU depot for storing and maintaining trains, and the capacity of the station building to accommodate passengers.

### 4.2 Notations

The definition of the sets and parameters mentioned in the railway hub network are summed in Tables 1 and 2.

**Table 1:** Notations of the sets.

Sets	Definition
K	set of trains, indexed by k, kϵK; the train type includes the departure train, the arrival train and the passing train, which are represented as $K_{\text{1}},\;K_{\text{2}},\;K_{\text{3}}$, $K=K_{\text{1}}\cup K_{\text{2}}\cup K_{\text{3}}$
V	set of nodes, indexed by v, vϵV
A	set of stations, indexed by a, aϵA, AϵV
B	set of directions, indexed by b, bϵB, BϵV
P	set of traffic zones, indexed by p, pϵP, PϵV
Z	set of virtual nodes, indexed by $\overset{o}{\underset{k}{z}}$, $\overset{d}{\underset{k}{z}}$, kϵK, ZϵV
G	set of tracks, indexed by g, gϵG; the set of tracks in station a is represented as $G_{a}$, aϵA
E	set of arcs, indexed by (i,j), i,jϵV, (i,j)ϵE
$E_{\text{1}}$	set of railway lines, indexed by (i,j), i,jϵA∪B, $\;E_{\text{1}}\epsilon E$
$E_{\text{2}}$	set of passenger travel routes, indexed by (p,a), pϵP, aϵA, $\;E_{\text{2}}\epsilon E$
$E_{\text{3}}$	set of virtual arcs, indexed by $\left(\overset{o}{\underset{k}{z}},a),\;(a,\;\overset{d}{\underset{k}{z}}\right)$, $\overset{o}{\underset{k}{z}},\;\overset{d}{\underset{k}{z}}\epsilon Z,\;a\epsilon A$, $\;E_{\text{3}}\epsilon E$

**Table 2:** Notations of the parameters.

Parameters	Definition
$o_{k}$	origin of train k, kϵK
$d_{k}$	destination of train k, kϵK
$c_{ij}$	train capacity of arc (i,j), $(i,j)\epsilon E_{\text{1}}\cup E_{\text{3}}$
$t_{ij}$	train running time of arc (i,j), $(i,j)\epsilon E_{\text{1}}\cup E_{\text{3}}$
$u_{g}$	train capacity of track g, gϵG
$t_{g}$	train operation time of track g, gϵG
$q_{pb}$	the number of passengers from traffic zone p to direction b, pϵP, bϵB
$t_{pa}$	travel time of passengers from traffic zone p to station a, pϵP, aϵA
$w_{\text{1}}$	per time cost of trains
$w_{\text{2}}$	per time cost of passengers
*h*	passenger capacity of a single train

Notably, in order to indicate that departure and arrival trains can be operated at any station within the hub, virtual nodes and arcs are set. For the departure train $k\epsilon K_{\text{1}}$, the virtual origin $\overset{o}{\underset{k}{z}}$ is built to connect all stations, and the arc $\left(\overset{o}{\underset{k}{z}},a\right)$ refers to the train can be operated in station a. For the arrival train $k\epsilon K_{\text{2}}$, the virtual destination $\;\overset{d}{\underset{k}{z}}$ is also built to connect all stations, and the arc $\left(a,\;\overset{d}{\underset{k}{z}}\right)$ refers to the train can stop in station a.

### 4.3 Formulation

The common approaches of the train routing problem include the Node-Arc and the Arc-Route formulation. The Node-Arc formulation focuses on the occupancy of nodes and arcs, which can detail the train routes. The Arc-Route formulation needs to construct candidate sets of train routes, which can simplify the solving methods. In this study, to comprehensively represent all possible routes and track allocation, the Node-Arc formulation is adopted instead of the Arc-Route formulation. Therefore, the decision variables are defined to represent arc usage and track occupancy. The formal definitions of these variables are listed in Table 3.

**Table 3:** Notations of decision variables.

Decision Variables	Definition
$\overset{k}{\underset{ij}{x}}$	Binary, {0,1}, represents the train k usage of arc (i,j), equals 1 if train k passes arc (i,j), and 0 otherwise, kϵK, $(i,j)\epsilon E_{\text{1}}\cup E_{\text{3}}$
$\overset{k}{\underset{g}{y}}$	Binary, {0,1}, represents the train k occupancy of track g, equals 1 if train k is operated at track g, and 0 otherwise, kϵK, gϵG
$r_{pab}$	Integer, represents the number of passengers boarding at station a to direction b, pϵP, aϵA, bϵB, $(p,a)\epsilon E_{\text{2}}$

Drawing on the relevant theories of multi-commodity network flow and combining the above decision variables, the optimization model for train routing problem in the railway hub considering multi-station collaboration and passenger demand is constructed. The objective function and constraints are stated as follows:

(1) The objective function

To minimize the total cost of trains and passengers, the objective function is represented as follows:


$$\begin{array}{c} min\sum_{k\epsilon K}(\sum_{(i,j)\epsilon E_{\text{1}}\cup E_{\text{3}}}\overset{k}{\underset{ij}{x}}t_{ij}w_{\text{1}}+\sum_{g\epsilon G}\overset{k}{\underset{g}{y}}t_{g}w_{\text{1}})+\sum_{(p,a)\epsilon E_{\text{2}},\;b\epsilon B}r_{pab}t_{pa}w_{\text{2}} \end{array}$$ (1)

In Eq. (1), the objective function includes two parts: the train cost and the passenger cost. For the first part, we aim to obtain the shortest train routes, so the train cost is calculated by the train running time on the railway lines and the operation time at tracks within stations multiplied by per time cost of trains. For the second part, we aim to make all passengers board at their nearest stations, so the passenger cost is calculated by the passenger travel time on the travel routes multiplied by per time cost of passengers.

(2) Constraints

The constraints focus on the capacity of the railway hub and passenger demand, which include flow balance constraints, no circle constraints, stop frequency constraints, the relationship between stop stations and train routes constraints, capacity of arcs constraints, capacity of tracks constraints, capacity of trains constraints, distribution of passengers constraints and decision variables constraints. The specific formula is represented as follows:

Constraints (2) are flow balance constraints, which are the conventional constraints for multi-commodity network flow. For any train kϵK, the total occupation of all arcs departing from its origin $o_{k}$ minus the total occupation of arcs arriving at it equals 1. The total occupation of arcs departing from its intermediate nodes equals that of arcs arriving at them. The total occupation of arcs departing from its destination $d_{k}$ minus the total occupation of arcs arriving at it equals −1.


$$\begin{array}{c} \sum_{j\epsilon A\cup B\cup VN,\;j=\;\;/\;i}\overset{k}{\underset{ij}{x}}-\sum_{j\epsilon A\cup B\cup VN,\;j=\;\;/\;i}\overset{k}{\underset{ji}{x}}=\left\{ \begin{array}{c} \text{1},\;i=o_{k}\\ \text{0},\;i=\;\;/\;o_{k}\;and\;i=\;\;/\;d_{k}\\ -\text{1},i=d_{k} \end{array}\right.\;,\;\forall i\epsilon A\cup B\cup Z,\;\forall k\epsilon K \end{array}$$ (2)

Constraints (3) and (4) are no circle constraints, which ensure that there are no circles in the train routes.


$$\begin{array}{c} \sum_{j\epsilon A\cup B\cup VN}\overset{k}{\underset{ij}{x}}=\text{1},\;\forall i\epsilon A\cup B\cup Z,\;k\epsilon K \end{array}$$ (3)

$$\begin{array}{c} \sum_{j\epsilon A\cup B\cup VN}\overset{k}{\underset{ji}{x}}=\text{1},\;\forall i\epsilon A\cup B\cup Z,\;k\epsilon K \end{array}$$ (4)

Constraints (5) and (6) are stop frequency constraints. Considering excessive stops would lead to frequent starts and stops of the train, not only increasing energy consumption but also occupying capacity and prolonging the time to pass through the hub. Therefore, we stipulate that a train can make at most two stops within the hub, and each stop can only be carried out on one track for departing and arriving operations.


$$\begin{array}{c} \text{1}\leq \sum_{g\epsilon G}\overset{k}{\underset{g}{y}}\leq \text{2},\;\forall k\epsilon K \end{array}$$ (5)

$$\begin{array}{c} \sum_{g\epsilon G_{a}}\overset{k}{\underset{g}{y}}\leq \text{1},\;\forall a\epsilon A \end{array}$$ (6)

Constraints (7) indicate the relationship between stop stations and train routes, which ensure that the stop stations of the train must be located on its route.


$$\begin{array}{c} \sum_{j\epsilon A\cup B\cup VN}\overset{k}{\underset{ij}{x}}+\sum_{j\epsilon A\cup B\cup VN}\overset{k}{\underset{ji}{x}}\geq \sum_{g\epsilon G_{i}}\overset{k}{\underset{g}{y}},\;\forall i\epsilon A,\;\forall k\epsilon K \end{array}$$ (7)

Constraints (8) are capacity of arcs constraints, which belong to the capacity constraint in multi-commodity network flow. This set of constraints ensures that the number of trains passing through any railway line within the decision-making period is less than or equal to the capacity of arcs.


$$\begin{array}{c} \sum_{k\epsilon K}\overset{k}{\underset{ij}{x}}\leq c_{ij},\;\forall (i,j)\epsilon E_{\text{1}}\cup E_{\text{3}} \end{array}$$ (8)

Similar to the previous constraints, constraints (9) are capacity of tracks constraints, which represent the receiving and dispatching capacity constraint of tracks at the station. It ensures that the number of trains departing and arriving on a certain track within the decision-making period is less than or equal to its receiving and dispatching capacity.


$$\begin{array}{c} \sum_{k\epsilon K}\overset{k}{\underset{g}{y}}\leq u_{g},\;\forall g\epsilon G \end{array}$$ (9)

Constraints (10) are capacity of trains constraints, which are coupling constraints between train decision variables and passenger decision variables, indicating that the total number of passengers from all traffic zones boarding at station a to direction b is less than the total passenger capacity of all trains departing from station a to direction b.


$$\begin{array}{c} \sum_{p\epsilon P}r_{pab}\leq \sum_{k\epsilon K|d_{k}=b}\overset{k}{\underset{g}{y}}h,\;\forall a\epsilon A,\;\forall b\epsilon B \end{array}$$ (10)

Constraints (11) are distribution of passengers constraints, which indicate the distribution of passengers from the traffic zone p to direction b among stations.


$$\begin{array}{c} \sum_{a\epsilon A}r_{pab}\leq q_{pb},\;\forall p\epsilon P,\;\forall b\epsilon B \end{array}$$ (11)

Constraints (12) are decision variables constraints, respectively represent the domains of the train and passenger decision variables.


$$\begin{array}{c} \overset{k}{\underset{ij}{x}},\overset{k}{\underset{g}{\;y}}\epsilon \{\text{0},\text{1}\},\;\forall k\epsilon K,\;\forall (i,j)\epsilon E_{\text{1}}\cup E_{\text{3}},\;\forall g\epsilon G \end{array}$$ (12)

$$\begin{array}{c} r_{pab}\epsilon N^{+}\;and\;\text{0}{\leq r}_{pab}\leq q_{pb},\;\forall p\epsilon P,\;\forall a\epsilon A,\;\forall b\epsilon B \end{array}$$ (13)

As for the objective function (1) and constraints (2–13) are linear, the model M1 can be regarded as a mixed integer linear programming model (MILP). The proposed MILP belongs to the class of NP-hard problems, the worst-case computational time grows exponentially with problem size in theory. However, as the model structure is similar to the classical Vehicle Routing Problem(VRP), which has been solved efficiently by many mature algorithms, when the problem scale is not too large, M1 can be solved by using commercial solvers. The GUROBI solver is equipped with various exact and heuristic algorithms, offering fast solution speed and high solution quality. Therefore, the Python programming language is used to call the GUROBI solver within the PyCharm Community software platform to solve model M1. The running environment is a laptop with an Intel(R) Core(TM) i7-9750H CPU at 2.60 GHz and 16GB of memory.

## 5. Case study

### 5.1 Data source

**Fig 2:**
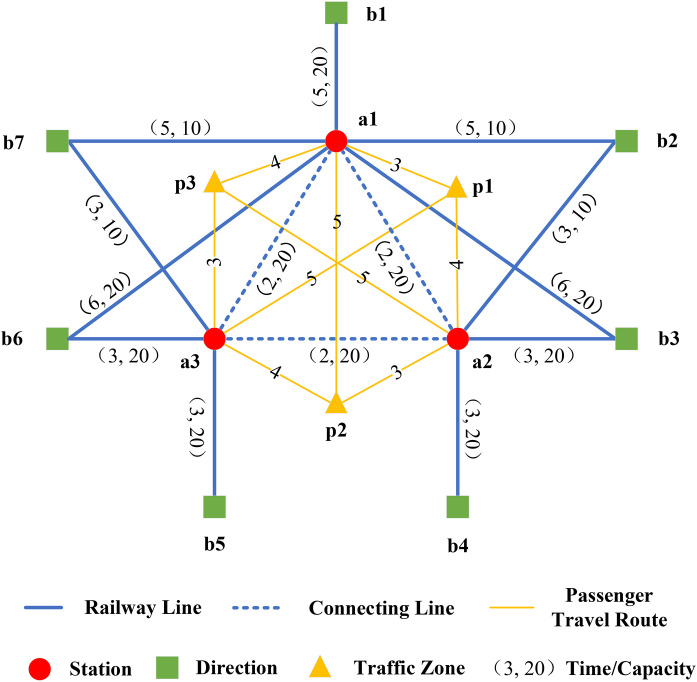
The network structure of the example railway hub.

Based on the current development status of railway hubs in China, it can be observed that the number of stations in a hub is generally no more than 3, and the number of connecting directions is generally no more than 10. For example, as one of the largest hubs in China, Zhengzhou High-Speed Railway Hub consists of three passenger stations: Zhengzhou Station, Zhengzhou East Station and Zhengzhou South Station, which connect 8 directions including Beijing, Jinan, Xuzhou, Hefei, Wuhan, Chongqing, Xi’an and Taiyuan railway lines.Therefore, this paper designs a calculation example based on the actual railway network scale. The example railway hub network designed in this section includes three passenger stations, seven directions and three traffic zones. The specific network structure is shown in Fig 2. All railway lines are single-direction double-track railways, and the line conditions of up and down directions are the same. The running time and capacity of each railway line, as well as the travel time of passengers on each travel route, are marked on the corresponding arcs in the figure. The operation time and capacity of tracks in each station are shown in Table 4. The operation time is directly proportional to the distance between the track and the main line. The train types and quantities of each direction within the hub are shown in Table 5, and the number of passengers traveling from each traffic zone to each direction is shown in Table 6. Based on actual railway operational data, the procurement cost of each train is approximately 150–200 million yuan. With an annual utilization of 140 days and factoring in depreciation expenses, the resulting operating cost amounts to roughly 1,000 yuan/minute [15,29]. In addition, passenger costs include direct time value and indirect costs, according to the average annual disposable income of urban residents, the travel time cost of passengers is approximately 10–15 yuan/minute [30,31]. Furthermore, in order to ensure that the influence of train cost and passenger cost on the objective function is at the same order of magnitude, after several sets of test experiments, per time cost of trains $w_{\text{1}}$ is set to 1,000 yuan/minute, and per time cost of passengers $w_{\text{2}}$ is 10 yuan/minute. The passenger capacity of all trains is set to 2,500 people per train.

**Table 4:** Operation time and capacity of tracks in the example stations.

Stations	Tracks				
	1	2	3	4	5
$a_{\text{1}}$	6, 1	6, 1	6, 1	6, 1	6, 2
$a_{\text{2}}$	6, 1	6, 1	6, 1	6, 2	6, 2
$a_{\text{3}}$	6, 1	6, 1	6, 1	6, 2	6, 2

**Table 5:** Number of trains of all directions in the example hub.

Directions	$b_{1}$	$b_{2}$	$b_{3}$	$b_{4}$	$b_{5}$	$b_{6}$	$b_{7}$
departure trains	2	2	2	2	2	2	2
arrival trains	2	2	2	2	2	2	2
passing trains	b1-b3:2 b1-b4:2 b1-b5:2 b1-b6:2	b2-b7:2	b3-b1:2 b3-b6:2	b4-b1:2	b5-b1:2	b6-b1:2 b6-b3:2	b7-b2:2

**Table 6:** Number of passengers of all traffic zones in the example hub.

Directions	$b_{1}$	$b_{2}$	$b_{3}$	$b_{4}$	$b_{5}$	$b_{6}$	$b_{7}$
$p_{\text{1}}$	3000	3000	3000	3000	3000	3000	3000
$p_{\text{2}}$	3000	3000	3000	3000	3000	3000	3000
$p_{\text{3}}$	4000	4000	4000	4000	4000	4000	4000

### 5.2 Optimized results

By inputting the basic data from the previous section into the optimization model M1. The Python language is used to call the GUROBI solver to obtain the optimization solution, with a solution time of 3.26 seconds.

The objective function value is calculated to be 21,396 thousand yuan, of which the train cost is 396 thousand yuan, including 332 thousand yuan for the train running cost on the railway lines and 64 thousand yuan for the train operation cost on the tracks. The passenger cost is 21,000 thousand yuan. The train routes and the operation tracks they stop at are shown in Table 7, and the passenger travel routes are shown in Table 8.

**Table 7:** Optimized train routes of the example hub.

No.	Train routes	Tracks	Stations	No.	Train routes	Tracks	Stations
1	o1-a1-b1	g3	a1	30	b1-a1-a2-b3	g4	a1
2	o2-a1-a2-b2	g4	a1	31	b1-a1-a2-b4	g2	a1
3	o3-a2-b3	g6	a2	32	b1-a1-a3-a2-b4	g11	a3
4	o4-a3-a2-b4	g12	a3			g3	a1
		g6	a2	33	b1-a1-a3-b5	g11	a3
5	o5-a2-a3-b5	g7	a2			g1	a1
6	o6-a3-b6	g11	a3	34	b1-a1-a3-b5	g15	a3
7	o7-a3-b7	g12	a3			g4	a1
8	o8-a1-b1	g3	a1	35	b1-a1-a3-b6	g1	a1
9	o9-a2-b2	g7	a2	36	b1-a1-a3-b6	g2	a1
10	o10-a2-b3	g7	a2	37	b2-a2-a3-b7	g14	a3
11	o11-a2-b4	g6	a2			g7	a2
12	o12-a2-a3-b5	g7	a2	38	b2-a2-a1-a3-b7	g9	a2
13	o13-a3-b6	g13	a3			g1	a1
14	o14-a1-a3-b7	g4	a1	39	b3-a2-a3-b6	g6	a2
15	b1-a1-d15	g2	a1	40	b3-a2-a3-b6	g6	a2
16	b2-a2-d16	g8	a2	41	b3-a2-a1-b1	g3	a1
17	b3-a2-d17	g6	a2	42	b3-a2-a1-b1	g4	a1
18	b4-a2-d18	g8	a2	43	b4-a2-a1-b1	g8	a2
19	b5-a3-d19	g12	a3	44	b4-a2-a1-b1	g7	a2
20	b6-a3-d20	g13	a3	45	b5-a3-a1-b1	g12	a3
21	b7-a3-d21	g13	a3	46	b5-a3-a1-b1	g11	a3
22	b1-a1-d22	g2	a1	47	b6-a3-a1-b1	g1	a1
23	b2-a2-d23	g8	a2	48	b6-a3-a1-b1	g4	a1
24	b3-a2-d24	g8	a2	49	b7-a3-a2-b2	g13	a3
25	b4-a2-d25	g8	a2			g9	a2
26	b5-a3-d26	g13	a3	50	b7-a3-a1-a2-b2	g11	a3
27	b6-a3-d27	g13	a3			g13	a1
28	b7-a3-d28	g12	a3	51	b6-a3-a2-b3	g12	a3
29	b1-a1-a2-b3	g1	a1	52	b6-a3-a2-b3	g11	a3

**Table 8:** Optimized passenger travel routes of the example hub.

Traffic Zones	Stations	Directions	Numbers
p1	a1	b1	3000
p1	a1	b2	3000
p1	a1	b3	3000
p1	a1	b4	3000
p1	a1	b5	3000
p1	a1	b6	3000
p1	a1	b7	3000
p2	a2	b1	3000
p2	a2	b2	3000
p2	a2	b3	3000
p2	a2	b4	3000
p2	a2	b5	3000
p2	a2	b6	3000
p2	a3	b7	3000
p3	a3	b1	4000
p3	a3	b2	4000
p3	a3	b3	4000
p3	a3	b4	4000
p3	a3	b5	4000
p3	a3	b6	4000
p3	a3	b7	4000

Based on the train routes and passenger travel routes in Tables 7 and 8, the following conclusions can be drawn: ① The routes of most trains are the shortest routes in the railway hub network, demonstrating the effectiveness of the optimization model M1 in limiting train operation costs; ② All passengers can reach the nearest stations to travel to their target directions, indicating that the optimization model M1 can not only optimize train routes but also better meet passenger demand; ③ Trains with the same origin-destination may have different routes and stop stations. For instance, No. 49 and 50 trains are both passing trains from b7 to b2, with various routes b7-a3-a2-b2 and b7-a3-a1-a2-b2, and stops at a2, a3 and a1, a3 respectively. This shows that the optimization model M1 can flexibly arrange the division of labor plan based on the track utilization rate of stations and the travel demand of nearby passengers; ④ Eight trains stop twice within the hub, proving that the optimization model M1 can take into account the passenger demands of multiple traffic zones; ⑤ The three passenger stations in the hub each undertake 20 trains, which indicates that the division of labor plan can effectively coordinate the capacity of all stations within the hub and balance the operation burden among stations.

### 5.3 Discussion

To further illustrate the effectiveness of the optimization method, this section will compare the optimization scheme with two other schemes, namely the fixed scheme and the stop-once scheme.

(1) Comparison with the fixed scheme.

The fixed scheme is a commonly used division mode in current railway hubs. Its characteristic is that each passenger station only receives and dispatches the trains of the nearest connecting direction. This division plan is relatively clear and conducive to train scheduling for the railway department.

The train routes are allocated based on the principle of the shortest path, and the travel routes of passengers are allocated under the passenger capacity constraints. The costs of each item in the objective function under the fixed scheme are calculated and compared with those in the optimized scheme. The results are shown in Table 9.

**Table 9:** Comparison between the cost of fixed scheme and optimization scheme (thousand yuan).

Scheme	Total	Train	Train running	Track operation	Passenger
Fixed	21418	418	348	70	21000
Optimization	21396	396	332	64	21000

The comparison results in Table 9 lead to the following conclusions: ① Compared with the fixed scheme, the total cost of the optimization scheme decreased by 0.1%, among which the train cost decreased by 5.3%, while the passenger cost remained unchanged. This indicates that the optimization scheme can effectively reduce the railway operation cost on the basis of meeting the passenger demand; ② Compared with the fixed scheme, the train running cost decreased by 4.6% and the track operation cost decreased by 8.6%. This shows that the optimization scheme can simultaneously optimize the train routes and stop plans.

**Fig 3:**
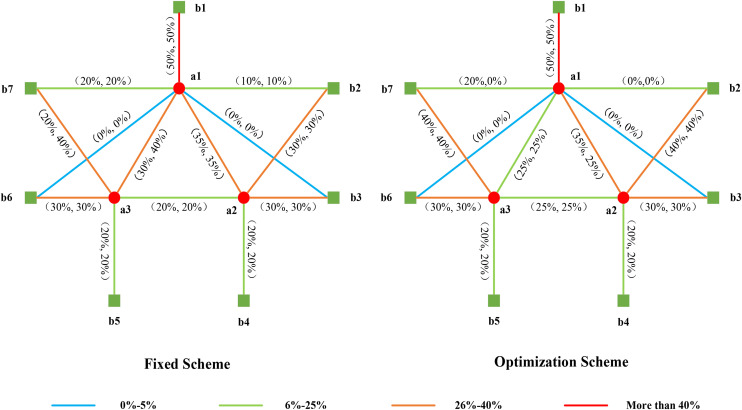
Comparison between arc capacity utilization rates of fixed and optimization scheme.

**Fig 4:**
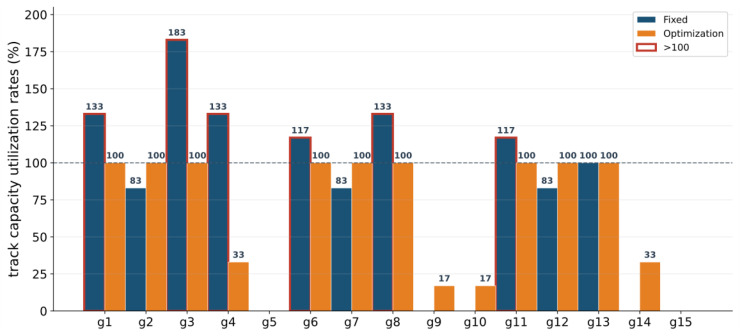
Comparison between track capacity utilization rates of fixed and optimization scheme.

To further refine the analysis of the optimization effect, focusing on the railway lines, the analysis is conducted from the perspectives of line capacity utilization rate and track capacity utilization rate, as shown in Figs 3 and 4 and Table 10.

**Table 10:** Track capacity utilization rates of fixed and optimization scheme.

Track	Station	Fixed Scheme(%)	Optimization Scheme(%)
g1	a1	133	100
g2	a1	83	100
g3	a1	183	100
g4	a1	133	33
g5	a1	0	0
g6	a2	117	100
g7	a2	83	100
g8	a2	133	100
g9	a2	0	17
g10	a2	0	17
g11	a3	117	100
g12	a3	83	100
g13	a3	100	100
g14	a3	0	33
g15	a3	0	0
**Variance**		0.36	0.17

From the comparison results of the capacity utilization rate in Figs 3 and 4 and Table 10, the following conclusions can be drawn: ① The arc capacity utilization rate of the fixed scheme and the optimization scheme is not significantly different. The number of tight sections (capacity utilization rate over 40%) is one for both schemes, and the number of busy sections (capacity utilization rate between 26% and 40%) is six and five respectively. This indicates that the optimization scheme has alleviated the busy situation of some sections, such as [a1, a3]; ② The fixed scheme can only obtain a feasible solution by relaxing the track capacity constraints, with the maximum track capacity utilization rate reaching 183%, which proves that the load on the busy tracks in the fixed scheme is relatively large. The maximum capacity utilization rate in the optimized scheme is 100%, which reduces the tight capacity situation of the busy tracks and avoids the overloading of stations; ③ The variances of the track operation capacity utilization rate for the fixed scheme and the optimization scheme are 0.36 and 0.17 respectively, which indicates that the optimization scheme makes more reasonable and balanced use of tracks, which is conducive to maintaining the flexibility of the station operation.

(2) Comparison with the stop-once scheme.

**Fig 5:**
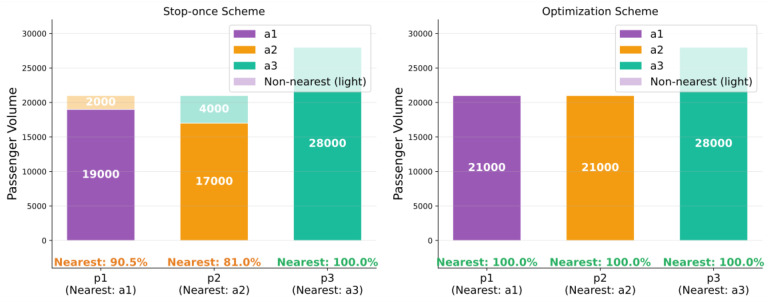
Comparison between passenger travel routes of stop-once and optimization scheme.

In practical railway operations, to reduce the train passing time within the hub, it is conventional to arrange trains to only stop once in the hub. However, this practice may be unable to fully accommodate the passenger demand of different urban areas. Specifically, the comparison of each cost item in the objective function is presented in Table 11, and the comparison of passenger travel routes is shown in Table 12 and Fig 5.

**Table 11:** Comparison between the cost of stop-once scheme and optimization scheme(thousand yuan).

Scheme	Total	Train	Train running	Track operation	Passenger
Stop-once	21980	380	326	54	21600
Optimization	21396	396	332	64	21000

**Table 12:** Passenger travel routes of the stop-once scheme and optimization scheme.

Stop-once Scheme				Optimization Scheme			
Traffic zones	Stations	Directions	Numbers	Traffic zones	Stations	Directions	Numbers
p1	a1	b1	3000	p1	a1	b1	3000
p1	a1	b2	2500	p1	a1	b2	3000
p1	a1	b3	3000	p1	a1	b3	3000
p1	a1	b4	2500	p1	a1	b4	3000
p1	a1	b5	2500	p1	a1	b5	3000
p1	a1	b6	3000	p1	a1	b6	3000
p1	a1	b7	2500	p1	a1	b7	3000
p1	a2	b2	500	p2	a2	b1	3000
p1	a2	b4	500	p2	a2	b2	3000
p1	a2	b5	500	p2	a2	b3	3000
p1	a2	b7	500	p2	a2	b4	3000
p2	a2	b1	3000	p2	a2	b5	3000
p2	a2	b2	2000	p2	a2	b6	3000
p2	a2	b3	3000	p2	a2	b7	3000
p2	a2	b4	2000	p3	a3	b1	4000
p2	a2	b5	2000	p3	a3	b2	4000
p2	a2	b6	3000	p3	a3	b3	4000
p2	a2	b7	2000	p3	a3	b4	4000
p2	a3	b2	1000	p3	a3	b5	4000
p2	a3	b4	1000	p3	a3	b6	4000
p2	a3	b5	1000	p3	a3	b7	4000
p2	a3	b7	1000				
p3	a3	b1	4000				
p3	a3	b2	4000				
p3	a3	b3	4000				
p3	a3	b4	4000				
p3	a3	b5	4000				
p3	a3	b6	4000				
p3	a3	b7	4000				

Based on the comparison results in Tables 11 and 12 and Fig 5, the following conclusions can be drawn: ① Compared with the stop-once scheme, the total cost of the optimization scheme decreased by 2.7%, among which the train cost increased by 4.2% while the passenger cost decreased by 2.8%. This indicates that although the optimization scheme slightly increased the train cost by stopping more times, it better balanced the passenger demand of multiple traffic zones, achieving the optimization effect of reducing the total cost; ② In the stop-once scheme, 71.4% of the passengers can board at the nearest stations to the target directions, while the optimization scheme enables all passengers to travel from the nearest stations, proving that the optimization scheme can better serve the passenger demand of different traffic zones within the city, which is conducive to improving the quality of railway passenger transport services.

## 6. Conclusions and future work

This paper takes railway hubs with multiple stations as the research subject, abstracting the division of labor problem among different stations as the train routing problem within the hub. To accommodate the travel demands of urban residents, multi-stop operations of trains are incorporated. By leveraging the multi-commodity network flow optimization theory and the Node-Arc modeling approach, a MILP optimization model for railway hubs is constructed. The objective function aims to minimize the sum of train cost within the hub and passenger cost travel from traffic zones to stations, with constraints including arc capacity, track capacity, and passenger capacity. Finally, a case study of a railway hub with three stations is designed to validate the effectiveness of the proposed model. The key conclusions are as follows:

(1) Compared with the fixed scheme, the optimization scheme reduces the total cost by 0.1%—with the total train cost decreasing by 5.3% while passenger remains unchanged. This demonstrates that the optimization scheme can effectively lower railway operation cost without compromising passenger demand and better balance the operational loads among different stations.
(2) Compared with the stop-once scheme, the optimization scheme cuts the total cost by 2.7%: train cost increases by 4.2% (attributed to additional stops), while passenger cost decreases by 2.8%. This indicates that despite a slight rise in track operation cost, the optimization scheme better addresses the passenger demand of multiple traffic zones.
(3) To ensure hub operational efficiency and service quality, railway departments should fully utilize the connecting lines within the hub to design more flexible division of labor schemes—thereby reducing train passing time and balancing operational loads. Additionally, appropriate multi-stop arrangements should be considered based on the distribution of passengers to better meet their mobility needs.

However, there are some limitations in this paper and the further work will focus on the following two points:

(1) This paper focuses on the outward travel demand of passengers within the hub but does not account for the transfer needs of transfer passengers. As the number of hub connecting directions increases in the future, rational accommodation of transfer passengers’ needs will become a critical optimization direction for division of labor.
(2) In the modeling process, some infrastructure conditions not central to this study (e.g., station throat capacity, EMU depot capacity) were simplified. Future work can refine these model details to make the optimization scheme more aligned with actual railway operations.
